# Mechanisms of germ cell survival and plasticity in *Caenorhabditis elegans*

**DOI:** 10.1042/BST20220878

**Published:** 2022-10-05

**Authors:** Wei Cao, Roger Pocock

**Affiliations:** Development and Stem Cells Program, Monash Biomedicine Discovery Institute and Department of Anatomy and Developmental Biology, Monash University, Melbourne, Victoria 3800, Australia

**Keywords:** apoptosis, *Caenorhabditis elegans*, germ cells, RNA splicing, starvation signaling

## Abstract

Animals constantly encounter environmental and physiological stressors that threaten survival and fertility. Somatic stress responses and germ cell arrest/repair mechanisms are employed to withstand such challenges. The *Caenorhabditis elegans* germline combats stress by initiating mitotic germ cell quiescence to preserve genome integrity, and by removing meiotic germ cells to prevent inheritance of damaged DNA or to tolerate lack of germline nutrient supply. Here, we review examples of germline recovery from distinct stressors — acute starvation and defective splicing — where quiescent mitotic germ cells resume proliferation to repopulate a germ line following apoptotic removal of meiotic germ cells. These protective mechanisms reveal the plastic nature of germline stem cells.

## Introduction

The primary objective of an animal is to transmit genetic material by reproduction. Animals not only need to assess and respond to the environment to maximize survival potential of their progeny, but also must manage internal stressors and regulate their reproduction accordingly. In response to stressors, the *Caenorhabditis elegans* germline arrests the mitotic germ cell cycle, likely in order to enable repair/synthesis pathways to operate [1–8]. In addition, meiotic germ cells undergo elevated programmed cell death (apoptosis) to remove cells that harbor DNA damage, or to provide biomaterial for oocyte growth under nutrient restricted conditions [1,2,5–12]. When a particular stressor is removed, quiescent mitotic germ cells can potentially resume proliferation and generate functional gametes. Such germline plasticity has been shown in studies of environmental stress (starvation) and genome instability (pre-mRNA splicing collapse) in *C. elegans* [2–4,10,13]. In this mini-review, we discuss stress induced by environmental factors and internal biological processes, how mitotic and meiotic germ cells respond to these stressors, and how fertility is re-enabled when stressors are removed.

## The *C. elegans* germline

**Figure 1.**
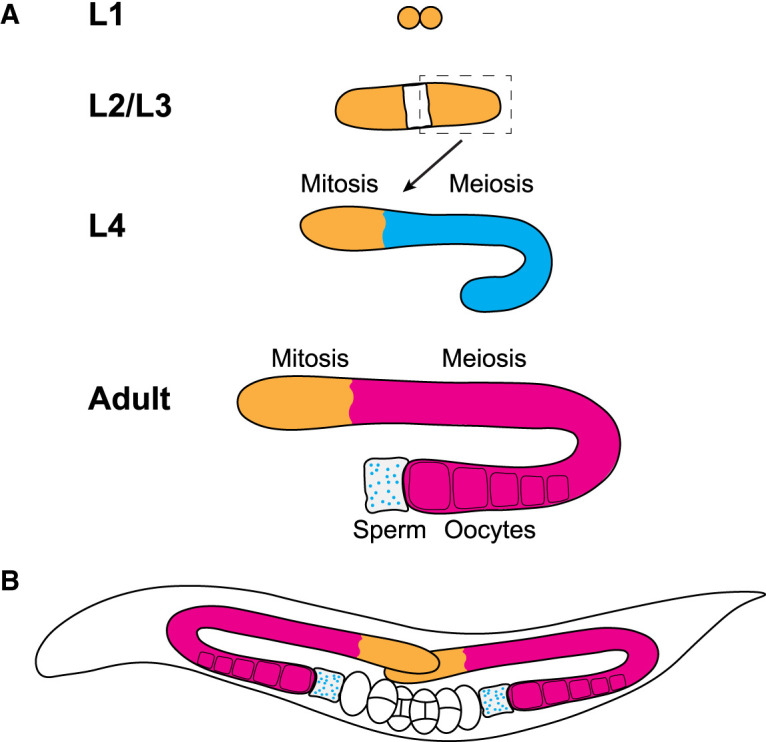
Post-embryonic development of the *C. elegans* hermaphrodite gonad. (**A**) Each germ line originates from two primordial germ cells that were generated in the embryo. These germ cells begin to proliferate from the mid first larval stage (L1) and continue to proliferate throughout larval and adult development. Meiosis commences during the late third (L3) and fourth larval stages (L4) when the germ lines generate sperm that are stored in the spermatheca. After entering adulthood, the germ line switches to produce oocytes. (**B**) Diagram of a *C. elegans* adult showing both gonad arms. Orange, proliferating germ cells; blue, spermatogenic meiotic cells; pink, oogenic meiotic cells; white, somatic cells.

Hermaphrodite *C. elegans* germ lines originate from two embryonically derived primordial germ cells that are quiescent in early L1 larvae (Figure 1A) [14,15]. These cells commence proliferation in mid-L1 larvae and continue to proliferate throughout larval development and early adulthood to populate the germline (Figure 1A) [14–16]. Over three days the two primordial germ cells generate two adult germline arms containing ∼1000 germ cells each (Figure 1B) [15,17]. At the distal end of the germline, a somatic distal tip cell expresses Notch ligands that maintain adjacent GLP-1 Notch receptor expressing germ cells in mitosis [18,19]. A subgroup of these cells located at the most distal end are germline stem cells [20,21]. As cells move away from the distal end, they begin to differentiate [22]. From the late L3 larval stage to the mid L4 larval stage, germ cells differentiate into sperm before the germ line switches to oocyte generation [23,24]. Sperm generated at this stage are stored in spermatheca. Approximately 50% of meiotic germ cells in the oogenic germline are sacrificed by physiological apoptosis. It is proposed that this mechanism provides material for oocyte growth [25].

## Germline stressors

**Figure 2.**
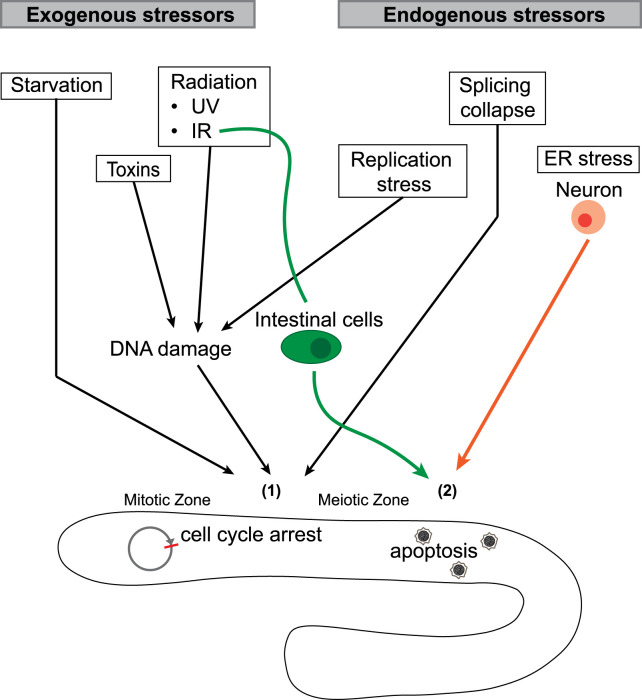
Schematic of stressors that regulate germ cell behavior. Exogenous stressors (starvation, radiation and toxins) and endogenous stressors (replication stress and splicing collapse) lead to mitotic cell cycle arrest and meiotic cell apoptosis (**1**). DNA damage pathways mediate radiation-, toxin- and replication stress-induced germ cell behavioral changes. In addition, intestinal cells respond to IR and enhance meiotic germ cell apoptosis (**2**) and ER stress in *C. elegans* sensory neurons promotes germ cell apoptosis (**2**).

Numerous environmental factors induce cellular and molecular changes to the mitotic cell cycle and meiotic cell fate in the *C. elegans* germline (Figure 2). These environmental factors include radiation (ultraviolet (UV) and ionizing (IR)), food availability, and toxins such as bisphenol A, tributyltin chloride, sodium arsenite and perfluorooctane sulfonate [2–11,26]. Most stressors damage germ cells by interfering with genome integrity, either directly or indirectly. UV results in DNA damage by exciting DNA bases with photons and directly causing DNA lesions, including cyclobutane pyrimidine dimers (CPDs), pyrimidine 6-4 pyrimidone photoproducts (6-4PPs), and their Dewar isomers [27,28]. Ionizing radiation directly causes double-strand breaks (DSB) and single-strand breaks (SSB), or even bistrand clusters that consist of both DSB/SSB and DNA base damage [29]. Additionally, both UV and IR indirectly cause DNA damage by inducing reactive oxygen species (ROS) [9,30]. Some toxins also cause DNA damage through indirect elevation of ROS and oxidative stress [6–8,11]. Other environmental factors induce germ cell apoptosis independently of DNA damage. These insults include abiotic stressors (oxidative, osmotic and heat stress) and pathogenic invasion [31,32]. Starvation also regulates the germ line through mechanisms independent of DNA damage and will be discussed separately.

In addition to environmental factors, cells experience chronic stress caused by biological processes. For example, the DNA replication machinery in germ cells continually needs to overcome exogenous or endogenous challenges to ensure the genome is replicated accurately and within required timeframes. DNA replication stress refers to the slowing or stalling of replication fork progression caused by errors induced by DNA damage [33–35]. Cells have an inherent ability to overcome physiological levels of DNA replication stress. To facilitate research on this phenomenon, studies induced replication stress using hydroxyurea (HU) to inhibit the enzyme ribonucleotide reductase, which inhibits deoxyribonucleotide synthesis and leads to stalled DNA replication forks [36–38]. The *C. elegans* DNA damage check point protein CLK-2/Tel2 was identified as essential for the DNA replication stress response [39]. Faithful RNA regulation is also critical for germ cell survival. For example, loss of RNA binding proteins such as CAR-1 and MINA-1 result in a marked increase in apoptosis [40,41].

Disruption of pre-mRNA splicing causes stress in cells by overloading mRNA clearance machinery and inhibiting the expression of proteins required to combat cell and molecular damage. Long-term disruption of splicing in *C. elegans* causes defects in the regulation of germ cell proliferation/differentiation and sex determination [42,43]. However, little is known regarding the direct effect of acute splicing collapse in germ cells, due to lethality of splicing mutants and lack of methods for robust temporal control of splicing factors. Our recent study (discussed in detail later) filled this knowledge gap using auxin-inducible protein degradation for conditional depletion of a pre-mRNA splicing factor MOG-7 in the germ line, which successfully induced global splicing defects [13].

Somatic tissues/cells can also regulate germ cell fate in response to both endogenous and exogenous stresses. Upon IR-induced stress, intestinal cells up-regulate germ cell apoptosis through PMK-1/p38 activated systemic stress signaling mediator (SYSM-1) expression and the transport of SYSM-1 into the germ line (Figure 2) [44]. In addition, ER stress in *C. elegans* sensory neurons promotes germ cell apoptosis via a highly conserved ER stress sensor, the ribonuclease inositol requiring protein-1 (IRE-1) (Figure 2) [45,46].

## How mitotic and meiotic germ cells respond to stress

To maintain genomic stability, mitotic germ cells undergo cell cycle arrest in response to stress induced by multiple factors, including toxins (sodium arsenite, perfluorooctane sulfonate), starvation, irradiation, and replication errors. For some stressors, little is known of the mechanisms through which cell cycle arrest is induced. However, DNA damage is the trigger for cell cycle arrest induced by irradiation and replication stress [5,47]. DNA damage induces arrest of mitotic germ cells with enlarged cellular and nuclear volume indicating excessive cellular growth [5]. DNA damage checkpoint proteins CLK-2/Tel2, HIM-7 and MRT-2/RAD1 control DNA damage induced mitotic arrest [5]. However, in most of these studies, mitotic cell arrest was assessed by the number of mitotic cells or the volume of mitotic germline compartments [5–8,39,48]. More refined analysis is needed to determine the changes in cell cycle length and the mechanism of mitotic arrest induced by different stressors.

To ensure the fidelity of genome inheritance, the germ line utilizes apoptosis to clear defective meiotic germ cells. In *C. elegans*, CED-9/Bcl-2, CED-4/Apaf-1 and CED-3/caspase comprise the core apoptotic machinery, and they mediate all germ cell apoptosis [49,50]. DNA damage induced by genotoxic agents (UV, IR and toxins) activates the core apoptotic machinery through CEP-1/p53 and two downstream transcriptional targets, EGL-1/BH3 and CED-13/BH3 [49,51,52]. Many DNA damage checkpoint proteins mediate DNA damage through the CEP-1 pathway, including RAD-51/RAD51, MRT-2/RAD1, HUS-1/HUS1 and CLK-2/Tel2 [5,6,8].

## Starvation and germline plasticity

*C. elegans* responds to starvation using multiple strategies during development [2,3,10,53,54]. Larvae respond to starvation by entering a developmental stage of arrest, known as dauer diapause, where animals reversibly arrest soma and germ line development, which resumes after refeeding [53,54]. Hermaphrodite L4 animals that encounter starvation can also slow germline development during the ‘germline starvation response’ (GSR) [2,3,10]. During this process, mitotic germ cells are quiescent, elevated apoptosis removes meiotic germ cells, and oocyte maturation is much slower than in well fed animals. All of these events cause germline shrinkage — leaving ∼35 distal germ cells.

It is not fully understood how starvation triggers the GSR. The starvation-sensing nuclear hormone receptor NHR-49/HNF-4α is required for differential expression of multiple fasting response genes upon food withdrawal, including genes involved in fatty acid oxidation and gluconeogenesis [55]. NHR-49 is also required for oogenic germ lines to respond to starvation by facilitating the establishment of the GSR and reproductive recovery [2]. As such, NHR-49 plays an important role in mediating environmental nutritional cues to the germ line.

In both male and hermaphrodite adults, mitotic germ cells respond to food removal rapidly by modifying the cell cycle [4]. Within 30 min of food removal, the number of germ cells in M phase reduces by more than half in early adults of both sexes, and reduces to zero after 3–4 h of starvation [4]. Interestingly, L4 hermaphrodite germ lines do not respond to starvation in this manner until they molt into adults, when the number of M-phase germ cells immediately falls to zero [4]. This again demonstrates that hermaphrodite germ line responses to stress are dependent on developmental stage. Cell cycle analysis by using 5-ethynyl-2-deoxyuridine (EdU) to mark cells in S phase shows that during starvation, germ cells slowly progress through S-phase and eventually arrest in G2 [4]. This cell cycle quiescence is accompanied by a gradual reduction in mitotic germ cell number [4].

It is not fully understood how starvation regulates the mitotic cell cycle. Germ cell quiescence induced by starvation does not require canonical food response pathways, including AMPK, insulin signaling, TGF-beta, neuropeptides, chemosensation or serotonin [4]. Mitotic quiescence induced by starvation is also different from that induced by DNA damage [4]. G2 arrested germ cells induced by starvation do not up-regulate tyrosine phosphorylation of cyclin-dependent kinase CDK-1, do not have enlarged nuclei, and do not require CEP-1/p53 [4,5,26,51]. Despite the essential role of GLP-1/Notch signaling in promoting mitotic proliferation, it is dispensable in starvation induced mitotic quiescence [4]. While removal of GLP-1/Notch signaling causes all germ cells to enter meiosis in fed animals, it does not affect mitotic germ cell fate during starvation [4].

Although the number of mitotic germ cells decreases in both male and hermaphrodite germ lines following starvation, the shrinkage of the whole germline only occurs in oogenic hermaphrodite germ lines [2–4,10]. Germline shrinkage is potentially the result of two processes — apoptosis and ovulation, which are both unique to the hermaphrodite. There is up to a 5-fold increase in apoptosis in the germ line during prolonged starvation, and germ cell corpse engulfment remains active [10]. Starvation induces germ cell apoptosis partially via the physiological LIN-35/Rb pathway, but not DNA-damage induced CEP-1/p53 pathway [10]. However, previous studies show contradictory results regarding the effect of apoptotic inhibition on germline shrinkage [2,10]. From the observation that feminized germ lines (through *fog-1* and *fog-2* mutations) do not decrease in size during the GSR, ovulation was proposed to be essential for germline shrinkage [10]. However, it is unknown if apoptosis increases normally during the GSR in these mutants. Further analysis is required to determine the contribution of these two processes in GSR induced germline shrinkage.

Gamete production continues during the GSR but is dramatically slowed — likely due to lack of biomaterial [3,10]. Upon starvation, each hermaphrodite germ line produces one oocyte at a time, in contrast with six to ten oocytes per germ line in fed animals [3]. Each oocyte only starts to grow after ovulation of the preceding oocyte [3]. If starvation commences before the completion of hermaphrodite spermatogenesis, the spermatogenic germ cells continue sperm production during starvation. As such, 5-day-starved wild-type animals still harbor ∼100 sperm in each gonad arm, which is similar to the number of sperm generated by fed animals [10]. However, after a prolonged period of starvation, very few sperm are observed, indicating a mysterious mechanism that removes sperm from gonad arms [2].

Starved germ lines demonstrate remarkable plasticity [2–4,10]. Even after 30 days of starvation, a shrunken germ line can regenerate a new germ line resembling that of a fed young adult after 2–3 days of re-feeding [2]. These recovered germ lines can produce some self-progeny, and additional progeny when mated with males [2,10]. However, their ability to produce progeny reduces with longer starvation periods [2]. This is partly due to the gradual loss of functional sperm during starvation, and increasingly severe impacts on oogenic germ cell health over time [2,10]. Germline regeneration following refeeding is primarily driven by arrested G2 stage germline stem cells that are preserved during starvation [4]. After 4 h of re-feeding, all these quiescent germ cells enter/progress through M phase, and the rate of progression through S phase and G2 is restored [4]. This indicates that arrested germline stems cells preserve the ability to proliferate.

## How do germ cells respond to splicing collapse?

Pre-mRNA splicing is an essential, conserved process that generates mature mRNA from nascent pre-mRNA. It is a complex process that involves binding of the spliceosome to splice sites, catalyzing the splicing reaction, and the recycling of spliceosome components [56,57]. Dysfunction of the splicing machinery could cause global endogenous stress in germ cells via multiple processes. The accumulation of pre-mRNA or mis-spliced mRNA might cause an overload of the mRNA decay system. Conversely, inefficient production of mRNA may lead to reduced cell cycle protein expression, generation of dysfunctional proteins and possibly proteotoxicity caused by incomplete splicing, and genotoxicity caused by lack of proteins required to protect the genome. These wide-ranging detrimental effects caused by splicing collapse provides a tool for examining germline plasticity under conditions of impaired transcriptome integrity.

In a reverse genetic screen, we discovered a splicing factor, MOG-7, that is essential for germline development [13]. The role of MOG-7 in splicing was suggested by its conserved functional domains and verified by proteomics, transcriptomics and splicing analysis [13]. MOG-7 encodes two functional domains — a pre-mRNA-splicing factor Ntr2 domain and a GC-rich sequence DNA-binding factor (GCFC) domain. In *Saccharomyces cerevisiae*, Ntr2 promotes Ntr1 recruitment into the intron lariat spliceosome (ILS) complex, which binds and activates Prp43 to induce spliceosome disassembly and release of the spliced intron and spliceosome components [58,59]. The human ortholog of MOG-7, C2ORF3, interacts with Prp43 and TFIP11 to control post-splicing intron turnover [60,61]. These studies in evolutionarily distant eukaryotes reveal that both functional domains of MOG-7 are associated with spliceosome disassembly. We identified EFTU-2/EFTUD2, PRP-8/PRPF8, PRP-19/PRPF19, SKP-1/SNW1 and STIP-1/NTR1 as MOG-7 interacting proteins *in vivo* [13]. These factors are essential components of the spliceosome, with STIP-1 controlling release of lariat introns and spliceosome disassembly after mature mRNA is released [59,62–65]. Remarkably, after just one hour of MOG-7 depletion we detected >17 000 retained introns, confirming a critical role for MOG-7 in RNA splicing [13].

Using conditional MOG-7 depletion specifically in the germ line, we found that pre-mRNA splicing is continuously required for germ cell development throughout the *C. elegans* lifespan [13]. MOG-7 depletion from early larval stages causes failure of germline development. In contrast, MOG-7 depletion from spermatogenesis prevents germ line transition into oogenesis, resulting in a germline that is arrested in a L4-like state, and when depleted after the sperm/oocyte switch, the germ line fails to produce functional oocytes or any progeny. This continuous MOG-7 requirement was confirmed by cell cycle analysis using EdU labeling. Following 6 h of MOG-7 depletion, reduced EdU labeling was observed, suggesting a reduction in mitotic proliferation [13].

We found that mitotic germ cells likely respond to splicing collapse by becoming quiescent. When MOG-7 was depleted from oogenic adult hermaphrodites, fewer germ cells progressed through S phase, suggesting cell cycle slowing/arrest [13]. The morphology of some germ cell nuclei that were not labeled by EdU suggest they arrested at the G2 stage [13]. However, after long term MOG-7 depletion, mitotic germ cells exhibit a variety of nuclear morphologies, suggesting that they arrest at different stages of the cell cycle [13].

Usually, meiotic germ cells respond to stress by increasing apoptosis. However, although MOG-7 depletion causes aberrant meiotic germ cell morphology, apoptosis is reduced [13]. This is likely due to inappropriate splicing of genes that encode proteins required for apoptosis. Interestingly, following MOG-7 depletion, germ cell nuclei in both mitosis and meiosis are larger, and the distance between germ cells is also increased [13]. Such germ cell enlargement resembles those that have experienced DNA damage [4,5].

## Germline plasticity following splicing inhibition

When MOG-7 is depleted from the L4 stage, we found that the resultant quiescent germ line can recover to produce viable progeny after MOG-7 expression is replenished, which presumably restores germ cell splicing. Like germ lines recovered from the GSR, two main processes are involved in this recovery: proliferation to generate new germ cells, and apoptosis for clearance of differentiated germ cells that were adversely affected by MOG-7 depletion.

The generation of new germ cells and gametes is essential for the germline to recover and produce progeny. Two days after restoring MOG-7 expression, new germ cells are generated, and the germ line resembles that of a young adult in size and has clearly defined mitotic and meiotic regions [13]. Labeling of S-phase nuclei after restoring MOG-7 expression also shows active mitotic proliferation. Collectively, we found that mitotic germ cells can resume proliferation and enter meiosis normally [13]. We also found that suppressing mitotic division by RNAi knockdown of *cdk-1*, which encodes a cyclin dependent kinase, completely prevents the germ line from producing progeny after restoring MOG-7 expression [13]. This indicates that differentiated oogenic germ cells that experienced splicing collapse cannot generate viable progeny.

Mitotic germ cells did not fully recover their function immediately after resuming proliferation. After MOG-7 expression is restored, the germline first erroneously generates sperm before producing oocytes [13]. Whether these are functional sperm is an open question. It has been shown that partial knockdown of spliceosome components by RNAi causes masculinization of the germ line, which we also found with *mog-7* RNAi [13,42]. Hence, a possible explanation is that inefficient splicing during early stages of MOG-7 recovery delays the production of proteins that are required for the sperm/oocyte switch.

Since differentiated oogenic germ cells affected by MOG-7 depletion cannot generate progeny, they need to be removed from the germ line. After MOG-7 expression was restored, we observed a burst of apoptosis at hour 18 that lasted for ∼12 h [13]. This was followed by an increase in progeny production in the third day of recovery, suggesting that removing defective germ cells facilitates recovery of fecundity. Using *ced-3* RNAi knockdown, we confirmed that apoptosis is essential for reproduction following MOG-7 restoration [13]. Since the inhibition of apoptosis does not affect mitotic germ cell proliferation or the generation of new gametes, the role of apoptosis here is likely to allow newly generated oocytes to access the spermatheca and sperm. Due to mis-splicing of apoptotic pathway genes during MOG-7 depletion, germ cells could not be cleared by apoptosis until MOG-7 expression was restored. The burst of apoptosis during recovery provided an opportunity to investigate the nature of the damage caused by splicing collapse. We found that LIN-35/Rb, CEP-1/p53, and EGL-1/BH3 are all partially required for the apoptotic removal of defective germ cells following MOG-7 recovery [13]. This suggests that MOG-7 depletion can cause both physiological and DNA damage apoptotic signals that are possibly dependent on the developmental stage of germ cells that encounter splicing collapse.

## Summary

**Figure 3.**
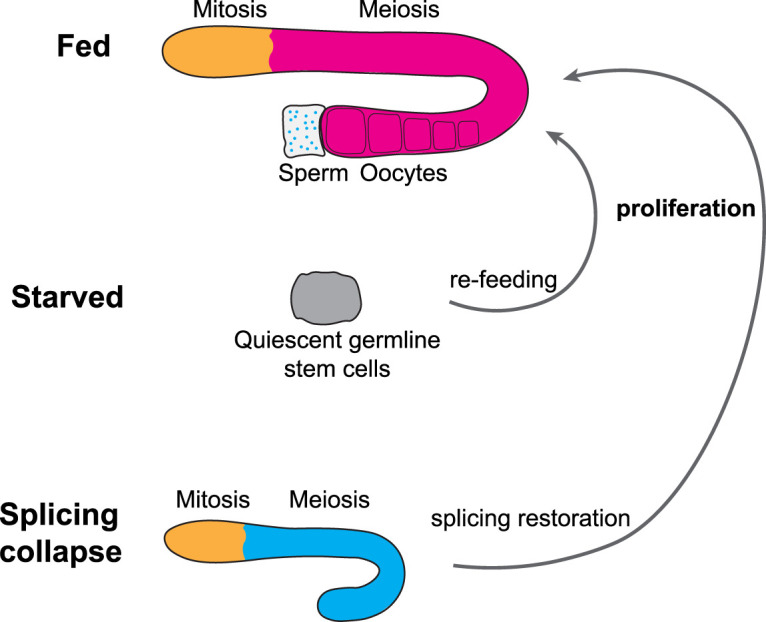
Schematic of germline regrowth after temporal starvation and splicing collapse. After prolonged starvation, hermaphrodite L4 germ lines shrink due to loss of meiotic germ cells via apoptosis and slowed ovulation, with only quiescent germline stem cells remaining. Splicing collapse pauses/slows the development of hermaphrodite L4 germ lines with mitotic germ cell becoming quiescent. These quiescent germ cells can recommence proliferation and generate a new germline after re-feeding or restoration of splicing. Defective germ cells caused by splicing collapse are cleared by apoptosis after splicing is restored. Orange, proliferating germ cells; gray, quiescent germ cells; blue, spermatogenic meiotic cells; pink, oogenic meiotic cells.

Animals constantly encounter environmental and intrinsic challenges that affect genome/transcriptome integrity, and the ability to grow. For species survival, animals must maintain reproductive fidelity to ensure their genomic content is accurately inherited. To achieve this, when a challenge is experienced, mitotic germ cells arrest to preserve their integrity and function, and meiotic germ cells undergo apoptosis to prevent inheritance of aberrant DNA or to support oocyte growth. When the threat is resolved, the germ line can recover its function by restoring proliferation (Figure 3). Here, we have provided examples of how the *C. elegans* germline responds to starvation and splicing disruption that reveal the remarkable plasticity of the germline to enable faithful species propagation.

## Perspectives

- Understanding how germ cells respond to distinct stressors is important for understanding fertility.
- Mitotic germ cells respond to stress by becoming quiescent, and meiotic germ cells respond to stress by activating apoptosis. The *C. elegans* germline can resume reproduction after a stressor is removed.
- Further investigation is needed to decipher mechanisms through which germline stem cells induce and remain quiescent under different types of stress.

## Open Access

Open access for this article was enabled by the participation of Monash University in an all-inclusive *Read & Publish* agreement with Portland Press and the Biochemical Society under a transformative agreement with CAUL.

## Abbreviations

DSB: double-strand breaks
EdU: 5-ethynyl-2-deoxyuridine
GSR: germline starvation response
HU: hydroxyurea
IR: ionizing radiation
ROS: reactive oxygen species
SSB: single-strand breaks
UV: ultraviolet
